# Inhibition of CYP2B6 by Medicinal Plant Extracts: Implication for Use of Efavirenz and Nevirapine-Based Highly Active Anti-Retroviral Therapy (HAART) in Resource-Limited Settings

**DOI:** 10.3390/molecules21020211

**Published:** 2016-02-16

**Authors:** Nicholas E. Thomford, Charles Awortwe, Kevin Dzobo, Faustina Adu, Denis Chopera, Ambroise Wonkam, Michelle Skelton, Dee Blackhurst, Collet Dandara

**Affiliations:** 1 Division of Human Genetics, Department of Pathology & Institute of Infectious Disease and Molecular Medicine, Faculty of Health Sciences, University of Cape Town, Anzio Road, Observatory, Cape Town 7925, South Africa; THMNIC023@myuct.ac.za (N.E.T.); ambroise.wonkam@uct.ac.za (A.W.); michelle.skelton@uct.ac.za (M.S.); 2 School of Medical Sciences, University of Cape Coast, Cape Coast, PMB, Ghana; a.faustina@uccsms.edu.gh; 3 Division of Clinical Pharmacology, Faculty of Medicine and Health Sciences, University of Stellenbosch, Cape Town 7602, South Africa; charzos@yahoo.com; 4 International Center for Genetic Engineering and Biotechnology (ICGEB), Cape Town component, Faculty of Health Sciences, University of Cape Town, Anzio Road, Observatory, Cape Town 7925, South Africa; kd.dzobo@uct.ac.za; 5 Division of Medical Biochemistry, Faculty of Health Sciences, University of Cape Town, Anzio Road, Observatory, Cape Town 7925, South Africa; 6 Division of Medical Virology, Department of Pathology & Institute of Infectious Disease and Molecular Medicine, Faculty of Health Sciences, University of Cape Town, Anzio Road, Observatory, Cape Town 7925, South Africa; denis.chopera@uct.ac.za; 7 Division of Chemical Pathology, Department of Pathology & Institute of Infectious Disease and Molecular Medicine, Faculty of Health Sciences, University of Cape Town, Anzio Road, Observatory, Cape Town 7925, South Africa; dee.blackhurst@uct.ac.za

**Keywords:** herb-drug interactions, CYP450, time-dependent inhibition, reversible inhibition, recombinant human CYPs

## Abstract

Highly active antiretroviral therapy (HAART) has greatly improved health parameters of HIV infected individuals. However, there are several challenges associated with the chronic nature of HAART administration. For populations in health transition, dual use of medicinal plant extracts and conventional medicine poses a significant challenge. There is need to evaluate interactions between commonly used medicinal plant extracts and antiretroviral drugs used against HIV/AIDS. Efavirenz (EFV) and nevirapine (NVP) are the major components of HAART both metabolized by CYP2B6, an enzyme that can potentially be inhibited or induced by compounds found in medicinal plant extracts. The purpose of this study was to evaluate the effects of extracts of selected commonly used medicinal plants on CYP2B6 enzyme activity. Recombinant human CYP2B6 was used to evaluate inhibition, allowing the assessment of herb-drug interactions (HDI) of medicinal plants *Hyptis suaveolens*, *Myrothamnus flabellifolius*, *Launaea taraxacifolia*, *Boerhavia diffusa* and *Newbouldia laevis*. The potential of these medicinal extracts to cause HDI was ranked accordingly for reversible inhibition and also classified as potential time-dependent inhibitor (TDI) candidates. The most potent inhibitor for CYP2B6 was *Hyptis suaveolens* extract (IC_50_ = 19.09 ± 1.16 µg/mL), followed by *Myrothamnus flabellifolius* extract (IC_50_ = 23.66 ± 4.86 µg/mL), *Launaea taraxacifolia* extract (IC_50_ = 33.87 ± 1.54 µg/mL), and *Boerhavia diffusa* extract (IC_50_ = 34.93 ± 1.06 µg/mL). *Newbouldia laevis* extract, however, exhibited weak inhibitory effects (IC_50_ = 100 ± 8.71 µg/mL) on CYP2B6. *Launaea taraxacifolia* exhibited a TDI (3.17) effect on CYP2B6 and showed a high concentration of known CYP450 inhibitory phenolic compounds, chlorogenic acid and caffeic acid. The implication for these observations is that drugs that are metabolized by CYP2B6 when co-administered with these herbal medicines and when adequate amounts of the extracts reach the liver, there is a high likelihood of standard doses affecting drug plasma concentrations which could lead to toxicity.

## 1. Introduction

It has been shown unequivocally that antiretroviral therapy (ART) reduces HIV/AIDS mortality and prolongs life. However, attention has now shifted from reduction in mortality to improvement of the quality of life for people taking ART [1,2]. Across the world, there are huge disparities in health systems, thus, HIV/AIDS patients in different geographical regions receive different ART regimens mostly due to the different cost of the more than 30 antiretroviral (ARV) drugs approved by the US FDA. In resource constrained settings, the most commonly used ARV drugs include nevirapine (NVP) and efavirenz (EFV) which form the backbone of highly active antiretroviral therapy (HAART) regimens; thus, quite a number of patients use these two drugs during treatment [3,4]. Africa is a continent with a high prevalence of HIV/AIDS making up approximately 67% of the world’s population of HIV infected people [5]. Most ARV drugs used in Africa include EFV and NVP. EFV and NVP are non-nucleoside reverse transcriptase I (NNRTIs) inhibitors which act by inhibiting the reverse transcriptase enzyme. Both EFV and NVP are relatively affordable when compared to other ARV drugs [6]; hence, they are widely used in many African countries. EFV and NVP are substrates of the CYP2B6 enzyme [7,8] and plasma concentrations of these two ARVs are affected by changes in levels and activity of CYP2B6. CYP3A4 also metabolizes these two drugs but its contribution appears to be marginal [9]. EFV and NVP have been associated with a number of side effects including liver damage, nausea, vomiting, fever, diarrhoea, dyslipidemia and headache [10].

CYP2B6 enzyme metabolizes many commonly used drugs including bupropion, propofol and cyclophosphamide which should not be given together with EFV or NVP, to avoid drug-drug interactions. Among populations in health transition, especially those in resource-constrained countries, patients often make use of herbal medicinal plants in the management and treatment of several diseases in addition to use of conventional drugs [11]. HIV infection is an epidemic in resource constrained countries, particularly in sub-Sahara Africa; thus, it is inevitable that there is dual use of HAART and herbal medicinal plants. Herbal medicine is much cheaper and readily available in most settings.

There is substantial evidence supporting the potential of medicinal plants in combating diseases, especially in developing countries [12,13,14,15]. Although effective in the treatment of some diseases, the mechanisms of action for most herbal medicines remain largely unknown. A wide range of compounds (including phytochemicals) have been extracted from some of these medicinal plants so it is not inconceivable to be able to predict possible effects of herbal medicines on enzymes, such as CYP2B6, that metabolize commonly used drugs including EFV and NVP [16,17,18]. Some patients take herbal medicines and conventional medicines at the same time in a bid to get better more quickly, potentiating risk of herb–drug interaction (HDI) [19]. In spite of the wide use of herbal medications among HIV/AIDs patients taking EFV or NVP-based HAART, data on their pharmacokinetic and pharmacodynamics properties in humans remain lacking or scant [11,20]. Clinically significant interaction of herbs with prescribed medications via drug metabolism may worsen the health condition of patients if not detected earlier during treatment.

Cytochrome P450 enzymes (CYP450) are responsible for the metabolism of nearly 75% of phase I-dependent metabolism of clinically used drugs [21]. Relative expression of CYP2B6 ranges from 2% to 10% of the total hepatic content [22,23,24] and contributes to a significant proportion of drug metabolism [25]. Genetic polymorphism in CYP2B6 have been implicated in variations in the activity of the enzyme in metabolizing xenobiotics [26]. Inhibition of CYP2B6 by any drug, conventional and/or herbal, is likely to present in a similar manner as portrayed by polymorphisms that result in deficient enzyme activity. Thus, when herbal medicines are co-administered with ARV drugs, the possibility of herb-ARV drug interaction might be clinically significant and may lead to significant morbidity and mortality.

The purpose of this study was to evaluate the inhibition of CYP2B6 enzyme activity by selected medicinal plant extracts used by HIV patients to treat HIV-associated opportunistic infections. The medicinal plant extracts evaluated were extracted from *Hyptis suaveolens*, *Boerhavia diffusa*, *Newbouldia laevis*, *Launaea taraxacifolia* and *Myrothamnus flabellifolius*.

## 2. Results

This study provides us with an opportunity to understand the effects of five medicinal plants—*Hyptis suaveolens*, *Boerhavia diffusa*, *Newbouldia laevis*, *Launaea taraxacifolia* and *Myrothamnus flabellifolius*—on CYP2B6 activity. Data presented here can help to increase awareness among current and future patients of the potential effects of dual use of conventional drugs that are metabolized xenobiotic metabolising enzymes such as CYP2B6 and the above herbal medicinal plants. Our data shows that, indeed, for most of these herbal compounds, there is need for caution when co-administering them with other medications.

### 2.1. Inhibition Screening and IC_50_ Determination

**Figure 1:**
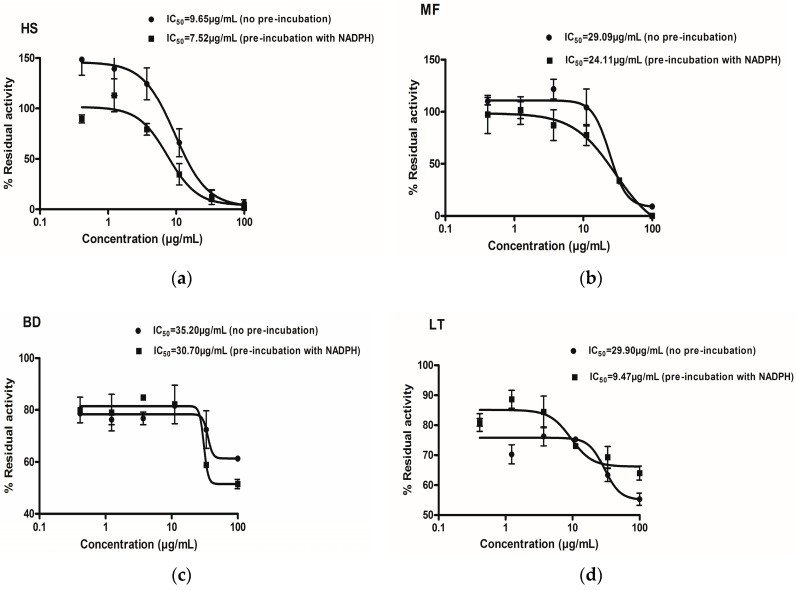
IC_50_ curve shift for Time Dependent Inhibition (TDI) determination. *Hyptis suaveolens* (HS) (**a**), *Boerhavia diffusa* (BD) (**c**), *Myrothamnus flabellifolius* (MF) (**b**) and *Launaea taraxacifolia* (LT) (**d**), at various concentrations were incubated with and without NADPH for 30 min. Percentage residual activity for no pre-incubation (closed circles) and 30 min pre-incubation (closed squares) is shown. Residual activity was calculated compared to control activity.

The potential of each medicinal plant extract to inhibit activity of recombinant human CYP2B6 is presented in Figure 1 and Table 1. Four of the medicinal plant extracts inhibited CYP2B6 activity in a concentration dependent manner. The potency of inhibition exhibited by the extracts was in the order of *Hyptis suaveolens* (HS) > *Myrothamnus flabellifolius* (MF) > *Boerhavia diffusa* (BD) > *Launaea taraxacifolia* (LT). *Newbouldia laevis* did not show any concentration dependent inhibitory effects.

The IC_50_ values for the medicinal plant extracts were determined as explained in the materials and methods section. The medicinal plant extracts inhibited CYP2B6 in the order *Hyptis suaveolens* (19.09 ± 1.16 µg/mL), *Myrothamnus flabellifolius* (23.66 ± 4.86 µg/mL), *Launaea taraxacifolia* (33.87 ± 1.54 µg/mL) and *Boerhavia diffusa* (34.93 ± 1.06 µg/mL) (Table 1). *Newbouldia laevis* indicated an IC_50_ of 100 ± 8.71 µg/mL, exhibiting very weak inhibitory effects on CYP2B6. Miconazole was used as a standard diagnostic inhibitor of CYP2B6 whose observed IC_50_ value of 0.55µM is in agreement with what has been reported elsewhere in literature [27].

**Table 1:** IC_50_ and IC_50_ fold shift due to incubation with or without Nicotinamide Adenine Dinucleotide Phosphate (NADPH).

Inhibitor	Conventional IC_50_ (µg/mL) (Mean ± SEM)	IC_50_ (no Pre-Incubation with NADPH) (µg/mL) (Mean ± SEM)	IC_50_ (Pre-Incubation with NADPH) (µg/mL) (Mean ± SEM)	Fold Shift (−IC_50_/+IC_50_)
*Newbouldia laevi*	100 ± 8.71	ND	ND	ND
*Hyptis suaveolens*	19.09 ± 1.16	10.60 ± 1.32	7.52 ± 1.20	1.40
*Launeae taraxacifolia*	33.87 ± 1.54	29.90 ± 1.32	9.47 ± 1.41	3.17
*Boerhavia diffusa*	34.93± 1.06	35.20 ± 2.86	30.70 ± 2.02	1.20
*Myrothamnus flabellifolious*	23.66 ± 4.86	29.09 ± 1.74	24.11 ± 1.24	1.21
Miconazole	0.53 ± 0.14	0.63 ± 0.15	0.72 ± 0.20	0.88

### 2.2. Prediction of in Vivo Herb-Drug Interaction for IC_50_

The % yield and concentration per dose was calculated as shown in Table 2. Since the intestinal absorption and plasma concentrations of each test compound are not known, and with the knowledge that herbal extracts have different bioavailability [28], the estimated bioavailable concentration was calculated using the % yield to give the soluble extract available in the GI tract.

**Table 2:** Calculation of herbal medicine concentration in the gut.

Herbal Extracts	% Yield	Recommended Herbal Dose (Single; mg)	Putative GIT Concentration (µg/mL)	Estimated Bioavailable Concentration (µg/mL)
*Newbouldia laevis*	14.66	200	800	117.28
*Hyptis suaveolens*	6.51	400	1600	104.16
*Launaea taraxacifolia*	10.40	200	800	83.3
*Boerhavia diffusa*	11.24	200	800	89.92
*Myrothamnus flabellifolius*	10.80	200	800	86.4

Note: GIT, Gastrointestinal tract, estimated bioavailable concentration = (% yield × putative GIT concentration)/100.

Using this assumption, the putative GIT concentration was calculated as shown in Table 2 and Table 3. The IC_50_ values obtained from the *in vitro* assay were then compared with the calculated estimated bioavailable concentration and predictions made. Based on the IC_50_ values, it was predicted that *Hyptis suaveolens*, *Boerhavia diffusa*, *Myrothamnus flabellifolius* and *Launaea taraxacifolia* were likely to cause *in vivo* inhibition of CYP2B6 and with potential HDI (Table 3). It was observed that IC_50_ values for *Hyptis suaveolens*, *Boerhavia diffusa*, *Myrothamnus flabellifolius* and *Launaea taraxacifolia* were approximately four times lower than the estimated bioavailable concentration and, therefore, adequate amounts may enter the hepatic portal vein to interact with CYP2B6. However, it was observed that NL had an IC_50_ value relatively similar to the estimated bioavailable concentration and, therefore, is not expected to enter the hepatic portal vein to interact with CYP2B6.

**Table 3:** *In vivo* prediction of HDI from *in vitro* for CYP2B6.

Herbal Extracts	Inhibitor Concentration (µg/mL)	IC_50_ (µg/mL)	Risk of HDI in the Gut *
*Newbouldia laevis*	117.28	100	Unlikely
*Hyptis suaveolens*	104.16	20.33	Likely
*Launaea taraxacifolia*	83.20	33.87	Likely
*Boerhavia diffusa*	89.92	34.93	Likely
*Myrothamnus flabellifolius*	86.40	23.66	Likely

Note: HDI, herb-drug interaction, inhibitor concentration = estimated bioavailable concentration (µg/mL), * the likelihood of a clinically relevant interaction when four of these herbal extracts are taken is based on the assumption that the % yield serves as the bioavailable fraction which was used in estimating the bioavailable concentration in the gut and also if there is complete absorption.

### 2.3. Time Dependent Inhibition (TDI) by IC_50_ Curve-Shift and Single Point NADPH Inhibition Screening Evaluation

The IC_50_ curve shift assay was used to evaluate the TDI potencies of the various extracts. A comparison was made between the IC_50_ values obtained with or without NADPH. The IC_50_-curve shift showing the potency of the plant extracts to cause TDI is shown in Figure 1.

**Figure 2:**
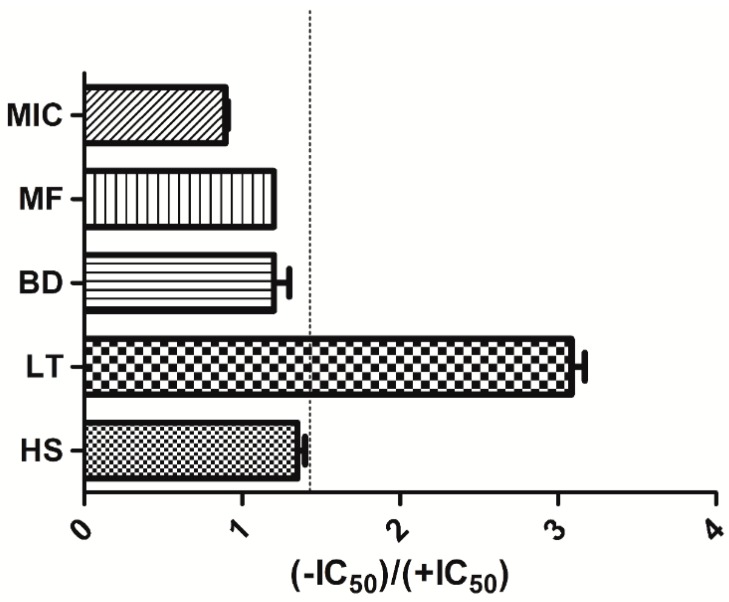
Time dependent inhibition (TDI) classification of medicinal plant extract incubation based on IC_50_ curve shift.

A fold decrease in IC_50_ was used to categorize a plant extract as a potential TDI. Extracts with an IC_50_ ratio of ≥1.5 were classified as positive TDIs as shown in Figure 2. Table 1 shows the shifted IC_50_ obtained for the various extracts. *Launaea taraxacifolia* showed TDI potency with an IC_50_ fold decrease of 3.17. However, *Hyptis suaveolens, Boerhavia diffusa* and *Myrothamnus flabellifolius* did not exhibit a TDI effect on CYP2B6.

Extracts of *Hyptis suaveolens* (HS), *Boerhavia diffusa* (BD), *Myrothamnus flabellifolius* (MF) and *Launaea taraxacifolia* (LT) on recombinant CYP2B6. The bars represent ratio of IC_50_ values from the no pre-incubation assays for the herbal extracts listed. Extracts that were >1.5 (dashed line) were classified as TDI candidates. Data shown are the mean ± SEM (*n* = 2).

**Figure 3:**
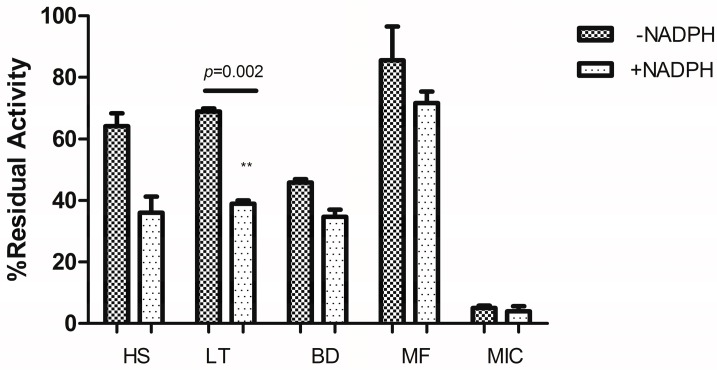
Inhibitory effects of 50 µg/mL of each extract after pre-incubation in the presence and absence of NADPH. ** Significant *p* value.

Specific concentrations of the plant extracts were pre-incubated with or without NADPH and the percentage residual activity was compared (Figure 3). Medicinal plant extracts that were pre-incubated with NADPH showed a reduction in the percentage activity of CYP2B6 compared to those without NADPH with a significant effect observed for the *Launaea taraxacifolia* extract indicating its time dependent inhibitory potency without the possibility of enzyme activity recovery.

*Hyptis suaveolens* (HS), *Boerhavia diffusa* (BD), *Myrothamnus flabellifolius* (MF) and *Launaea taraxacifolia* (LT) on recombinant CYP2B6 after pre-incubation with or without NADPH for 30 min. Percentage residual activities were plotted against respective fractions. Two-tailed unpaired *t*-test was used to compare the percentage residual activity of each fraction with *p* < 0.05 considered significant.

### 2.4. Relative Quantification of Selected Phenolic Compounds Using UPLC-MS

**Figure 4:**
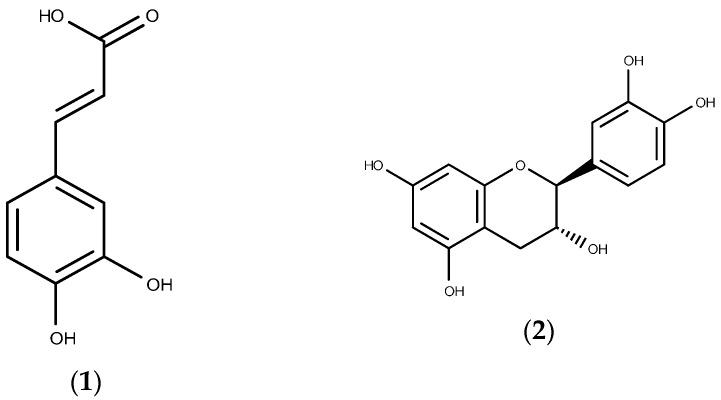

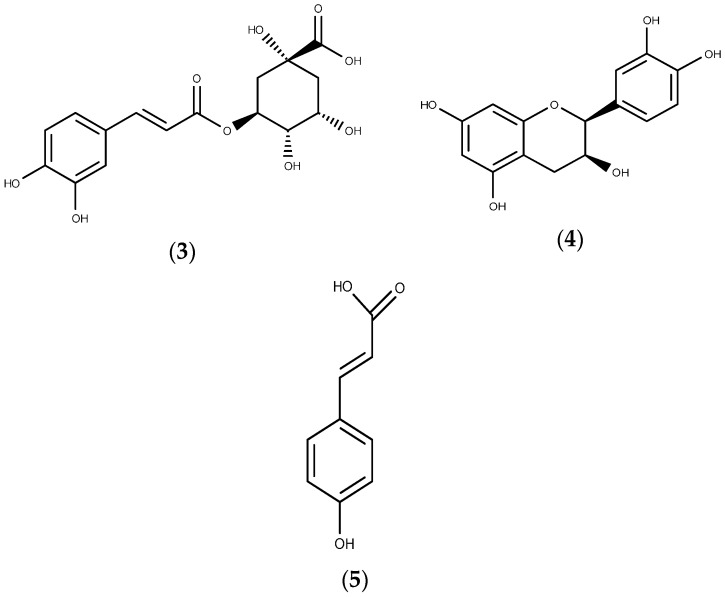
Structures of the phenolic compounds identified and quantified from crude herbal extracts (**1**) Caffeic acid; (**2**) Catechin; (**3**) Chlorogenic acid; (**4**) Epicatechin; (**5**) *p*-Coumaric acid.

The relative quantification of phenolic compounds after UPLC-MS was performed using XCMS data analysis software. Table 4 shows the relative quantification of the phenolic compounds analyzed using MS detection expressed in mg·kg^−1^ of original starting material used in the extraction, whereas Figure 4 shows structures for some of the identified compounds. *Hyptis suaveolens* had the highest content of chlorogenic acid (1665.33 ± 0.87 mg·kg^−1^) followed by *Launaea taraxacifolia* (1662.98 ± 2.10 mg·kg^−1^). *Launaea taraxacifolia* however contained the highest content of caffeic acid (998.59 ± 1.48 mg·kg^−1^) followed by *Hyptis suaveolens* (100.00 ± 0.97 mg·kg^−1^). Non-significant traces of epicatechin and catechin were found in all the medicinal plant extracts except *Myrothamnus flabellifolius*.

**Table 4:** Quantification of compounds identified in herbal extracts using MS detection.

Phenolic Compound	*Newbouldia laevis* (Mean ± SEM)	*Hyptis suaveolens* (Mean ± SEM)	*Launaea taraxacifolia* (Mean ± SEM)	*Boerhavia diffusa* (Mean ± SEM)	*Myrothamnus flabellifolius* (Mean ± SEM)
Catechin	Trace	Trace	Trace	1.40 ± 0.03	27.58 ± 0.10
*p*-Coumaric acid	7.54 ± 0.10	75.25 ± 0.10	751.41 ± 1.30	13.89 ± 0.10	39.13 ± 0.21
Caffeic acid	10.01± 0.01	100.00 ± 0.97	998.59 ± 1.48	10.59 ± 0.06	3.98 ± 0.01
Epicatechin	Trace	Trace	Trace	Trace	170.06 ± 0.39
Chlorogenic acid	166.77 ± 0.20	1665.33 ± 0.87	1662.98 ± 2.10	Trace	5.84 ± 0.10

Note: values are expressed as mg·kg^−1^ for four biological replicates ± SD.

## 3. Discussion

The use of medicinal plants for therapeutic purpose is a globally known phenomenon. When adverse effects are experienced or cure fails, like in the case of HIV infection and cancer, limited resources could be used to promote the use of herbal medicines. Thus, studies such as this one, which seek to scientifically validate the safety of herbal medicines, are imperative. Herbal medicine is being used for the treatment of a wide spectrum of disease conditions including cardiovascular and viral disease, cancer and diabetes [29]. There is increasing evidence to also show that there is concomitant use of herbal medicines with conventional medicines which leads to clinical complications resulting from herb–drug interactions [11,19,30]. Studies on the drug interaction potential of herbal medicines, especially those that have been in use for centuries, will help to create an awareness of the potential for complications [31,32]. It is also imperative that identification of the phytochemical constituents in these herbal medicines be performed to identify those likely to cause drug interactions. The advantage of phytochemical profiling is that similar drug interacting constituents could be identified in other related herbs that are used for therapeutic purposes and that necessary mechanistic studies are performed on them. Guidelines from the FDA relating to drug interaction and identification of compounds which interact with drug metabolizing enzymes and transporters is of high value since these compounds play a major role in affecting the pharmacokinetic and pharmacodynamics profiles of allopathic medications [33].

In this study, five herbal medicines commonly taken to treat and manage the effects of HIV/AIDS or its comorbidities by patients were evaluated for their ability to modulate activity of CYP2B6 *in vitro* using recombinant human CYPs. Crude extracts of the herbal medicines were used because since patients take these extracts in their crude from, it became important that the effect of the crude form is evaluated. The method of extraction used for this study was also performed to mimic the indigenous mode of extraction using water. The study showed that *Launaea taraxacifolia Hyptis suaveolens*, *Boerhavia diffusa* and *Myrothamnus flabellifolius* extracts caused a concentration-dependent inhibition in CYP2B6. The US FDA, EMA and pharmaceutical industries have published opinion documents and guidelines for the conduct of drug enzyme inhibition studies [34,35,36] which include reversible and time dependent inhibition profiles of new chemical entities (NCE). A reversible inhibition profile was thus conducted for the herbal extracts. It was observed that *Hyptis suaveolens*, *Myrothamnus flabellifolius*, *Launaea taraxacifolia* and *Boerhavia diffusa* had a reversible inhibitory effect on the activity of CYP2B6. *Newbouldia laevis,* however, did not show any significant inhibitory effects on the activity of CYP2B6 based on the concentration range assessed.

Based on the recommended dosage taken by patients, the GIT concentration was calculated and % yield used to estimate the bioavailable concentration (Table 2) and the possibility of causing HDI *in vivo* determined. Here, a comparison was made between the amount of extract in the gut and the IC_50_ concentrations obtained. If the amount in the gut is at least two-fold (2×) or more higher than the IC_50_, then it is expected that adequate amounts of the extract will reach hepatic CYP2B6 to cause clinically significant HDI [37]. It was observed that *Hyptis suaveolens*, *Myrothamnus flabellifolius*, *Launaea taraxacifolia* and *Boerhavia diffusa* were likely to cause HDI in the gut (Table 3). *Newbouldia laevis,* however, seems unlikely to cause an HDI in the gut (Table 3). Thus, patients taking *Hyptis suaveolens*, *Myrothamnus flabellifolius*, *Launaea taraxacifolia* and *Boerhavia diffusa* would need to exercise caution as these herbs are likely to inhibit CYP2B6 and could render drugs that are CYP2B6 substrates—which include efavirenz and nevirapine—toxic.

According to US FDA, EMA and pharmaceutical industries guidelines on the conduct of drug enzyme inhibition studies [34,36,38], NCE that show significant reversible inhibition should also be assessed for time dependent inhibitory (TDI) effects. The IC_50_ shift approach is one of the recommended methods for TDI assessment [39,40]. A significant shift towards the left in the inhibition curves after pre-incubation shows potency for TDI. The method was validated with miconazole which has not been known to show any TDI effects. According to Berry and Zhao [39], a fold shift or IC_50_-shift decrease of ≥1.5 is significant for a NCE to be called a TDI compound. In this study, therefore, this criterion was used to classify the herbal extracts as potential TDI candidates. *Launaea taraxacifolia* showed an IC_50_-shift decrease of 3.17 and was thus classified to be a potential TDI candidate. *Hyptis suaveolens*, *Myrothamnus flabellifolius* and *Boerhavia diffusa* showed fold decreases of 1.40, 1.21 and 1.20, respectively. The effect of NCE that causes TDI is that even if the usage of the compound is discontinued, there is permanent destruction of enzyme which requires *de novo* synthesis of the enzyme of interest. Potential TDI compounds asserts inhibitory effects through formation of more inhibitory metabolites or the irreversible inactivation of enzymes by metabolic products that form haem or protein adducts [41]. *Launaea taraxacifolia* thus having been classified as a potential TDI candidate in this study demonstrates potential in inhibiting CYP2B6. *Hyptis suaveolens*, *Myrothamnus flabellifolius* and *Boerhavia diffusa* exhibited a non-TDI effect suggesting the potential of these three herbs interacting reversibly with CYP2B6.

*Launaea taraxacifolia* has been used for centuries in West African countries such as Ghana and Nigeria as a vegetable and also for managing dyslipidemia and liver diseases [42] which are complications of HIV/AIDS, its comorbidities [43,44,45,46] and HAART [10,47,48,49]. Patients on HAART regimens containing EFV and NVP are likely to experience adverse drug effects from *Launaea taraxacifolia* when used together. In resource-limited settings, *Launaea taraxacifolia* is a commonly used vegetable; thus, herb–drug interactions are highly likely to be reported.

Although the study analyzed effects of the crude herbal extracts as taken by patients and did not evaluate the effects of the individual phytochemical constituents or fractions, UPLC-MS was used to quantify some phenolic compounds known for their therapeutic effects and some that have been implicated in CYP inhibition. Quantification of catechin, *p*-coumaric acid, caffeic acid, epicatechin and chlorogenic acid was performed with known standards and analyzed using XCMS data analysis software. Extract from *Launaea taraxacifolia* showed high content of chlorogenic acid (1662.98 mg·kg^−1^) and caffeic acid (998.59 mg·kg^−1^) (Table 4) with though these were also present in *Hyptis suaveolens*, *Myrothamnus flabellifolius* and *Boerhavia diffusa* also contained varying amounts. Caffeic acid [50] and chlorogenic acid [51,52] have been implicated in the inhibition of CYP450 activity *in vitro*. It is therefore postulated that the inhibitory effects observed on CYP2B6 in the study could be via the effects of these phenolic compounds. Other phenolic compounds—for example catechin, epicatechin [53] and *p*-coumaric acid [54], known for their antioxidant and anti-inflammatory effects—were also found in the extracts which also explains some of their therapeutic uses.

## 4. Materials and Methods

### 4.1. Chemicals and Reagents

Miconazole nitrate was purchased from Sigma-Aldrich (St. Louis, MO, USA). Black costar 96 well plates were obtained from Thermo Fischer Scientific (Pittsburgh, PA, USA). Vivid^®^ CYP450 Blue Screening Kit and Vivid^®^ Substrate, 7-benzyl-oxymethyloxy-3-cyanocoumarin, (BOMCC) were purchased from Life Technologies, (Grand Island, NY, USA). Standard for epicatechin, catechin, chlorogenic acid, caffeic acid and *p*-coumaric acid for identification of constituents of herbal extracts were purchased from Chromadex (Wesel, Germany). Purified water (double-distilled and deionized) was obtained from Millipore (Bedford, MA, USA).

### 4.2. Plant Material

The following plants *Hyptis suaveolens* (UCC/BS/687), *Boerhavia diffusa* (UCC/BS/688), *Newbouldia laevis* (UCC/BS/689) and *Launaea taraxacifolia* (UCC/BS/690), Table 5, used in the study were obtained and authenticated by botanists from the University of Cape Coast and samples were kept in the herbarium in the department of Biological sciences with voucher numbers as indicated. *Myrothamnus flabellifolius*, Table 5, was obtained from Zimbabwe and authenticated by a botanist. Leaves of the plants were air dried and made into a powder using mortar and pestle. Ethical approval was obtained from the University of Cape Town Human Research Ethics committee with number HREC REF: 826/2014.

**Table 5:** Herbal plants and their medicinal value.

Plant Species	Commonly Found African Countries	Purported Medicinal Value
*Newbouldia laevis*	Ghana, Togo, Nigeria, Congo	Anti-malaria, immune booster, anti-bacterial, anti-fungal
*Hyptis suaveolens*	Ghana, Togo, Nigeria, Congo, Benin, Guinea	Anti-bacterial, anti-fungal, anti-malaria, anti-cholesterol
*Launaea taraxacifolia*	Ghana, Togo, Nigeria, Congo, Benin, Guinea, Cote d’Ivoire	Anti-bacterial, anti-fungal, anti-malaria, anti-cholesterol, urinary infections, anti-diabetic
*Boerhavia diffusa*	Ghana, Togo, Nigeria, Congo, Benin, Guinea, Cote d’Ivoire, South Africa	Anti-bacterial, hepatoprotective, anti-nociceptive
*Myrothamnus flabellifolious*	Zimbabwe, Botswana, South Africa, Uganda, Egypt	Anti-viral, immune booster

### 4.3. Extraction of Plant Material

A sample of 10 g of leaves from each plant was prepared in 100 mL of distilled water and heated for one hour at 60 °C to mimic the indigenous mode of extraction. The material was allowed to extract for 72 h at room temperature during which supernatant was decanted every 24 h and the solid residue reconstituted in the same volume of purified water for the extraction process to be repeated. The supernatants were pooled and centrifuged (14,000× *g*, 10 min) and filtered using filter paper (8 µm, Whatman International LTD, Maidstone, UK). The filtrates were freeze-dried using a Virtis sentry freeze dryer (the Virtis Company, NC, Gardiner, NY, USA) and the resulting powders were weighed. The dried extracts were stored in an airtight container and stored at −20 °C until needed for use.

### 4.4. Screening for Inhibition

The medicinal plant extracts were screened for inhibitory effects on CYP2B6 using a two point screening assay that used two concentrations of the extracts, 10 µg/mL and 100 µg/mL, respectively. The assays were performed using the Vivid^®^ CYP450 Screening Kits (Life Technologies, Grand Island, NY, USA) with the protocol provided by the supplier. The Vivid^®^ CYP450 screening kits are designed to assess the metabolic activity and inhibition of the predominant human CYP450 isozymes involved in hepatic drug metabolism by the use of 7-benzyl-oxymethyloxy-3-cyanocoumarin (BOMCC) as a probe substrate. Briefly, the two concentrations of medicinal plants extracts mentioned above were pre-incubated with a mixture of CYP2B6 BACULOSOME plus reagent and regeneration system (consisting of glucose-6-phosphate and glucose-6-phosphate dehydrogenase) in Vivid^®^ reaction buffer I (Master pre-mix) in a black Costar 96-well plate for 20 min at 37 °C. The reaction was initiated by adding a mixture of reconstituted BOMCC and NADP^+^ in Vivid^®^ reaction buffer I and incubated for 30 min at 37 °C. The reaction was stopped by adding ice-cold 20% Tris base/80% acetonitrile (ACN). The activity of the enzymes was monitored by measuring the formation of fluorescent metabolite at an excitation and emission wavelength of 405/460 nm. Flourescence (SSN instruments, Set Point Technology, South Africa) was measured using a Varian Cary eclipse advanced reads software. The two point screening assay was followed by IC_50_ determination which included preparation of serial dilutions of medicinal plant extracts from a concentration of 100 µg/mL. A mixture of CYP2B6 BACULOSOME plus reagent and regeneration system in Vivid^®^ reaction buffer I (Master Pre-mix) in a black costar 96-well plate were pre-incubated for 20 min at 37 °C. The reaction was carried out in a similar manner to the two-point screening assay.

### 4.5. Time-Dependent Inhibition (TDI) Assessment Using the IC_50_ Curve-Shift

Determination of time-dependent inhibition (TDI) followed the same procedure as that of the IC_50_ determination with slight modifications. Medicinal plant extracts with a starting concentration of 100 µg/mL were serially diluted in duplicate six consecutive times in the black costar 96-well plate. The black Costar 96-well plate was divided into two halves; reaction mixture A (CYP2B6 BACULOSOME, regeneration system, NADP^+^ and Vivid^®^ reaction buffer I) was applied to the first half and reaction mixture B (CYP2B6 BACULOSOME, regeneration system and Vivid^®^ reaction buffer I) was added into the second half of the plate. The mixture was pre-incubated at 37 °C for 30 min. After pre-incubation, reconstituted BOMCC and NADP^+^ in Vivid^®^ reaction buffer I was added to each well to start the reaction. The mixture was incubated for an additional 30 min and the reaction was terminated by adding ice-cold 20% Tris base/80% ACN. The activity of the enzymes was monitored by measuring the formation of fluorescent metabolite at the same excitation and emission wavelength as in the IC_50_ determination. For single point NADPH screening, based on the shift in the IC_50_ curve, 50 µg/mL concentrations of the extracts were pre-incubated with or without NADPH for 30 min. The procedure for TDI by IC_50_ curve-shift was followed as explained above. This single point NADPH screening is carried out in order to assess the significance of the effect of pre-incubating with NADPH at a single concentration.

### 4.6. Relative Quantification of Selected Phenolic Compounds Using UPLC-MS

In order to identify and quantify constituents in the medicinal plant extracts, chromatographic separation was performed on a Waters Acquity UPLC system (Waters Corporation, Milford, MA, USA) coupled to a PDA detector and Synapt G2 (ESI negative) following the procedure described by Farag and Wessjohann [55]. Quantification of glycosylated derivatives of epicatechin, catechin, chlorogenic acid, caffeic acid and *p*-coumaric acid was calculated from calibration curves of epicatechin, catechin, chlorogenic acid, and caffeic acid and *p*-coumaric acid standards, respectively. Calibration curves for each reference compound ranged from 0.1 to 100 µg/mL.

### 4.7. Data Analysis

#### 4.7.1. IC_50_ Determination

The data generated was exported into an excel spreadsheet and the amount of metabolite formed at the various concentrations relative to the control (residual activity) was calculated using Equation (1)

% Residual Activity = (Test − Test blank)/(Control − Control blank) × 100
(1)

The percentage residual activity was plotted against the log transformed concentrations of the medicinal plant extracts and the miconazole (positive CYP2B6 inhibitor). A sigmoid curve was then fitted using non-linear regression analysis and the IC_50_ value was calculated using GraphPad Prism version 5.0 (GraphPad Software Inc., San Diego, CA, USA). IC_50_ values were calculated with the remaining enzyme activity and inhibitor concentration using Equation (2)

Y = 100 − [(100 × [I]^H^)/(IC_50_^H^ + [I]^H^)] (2)

Y: remaining CYP2B6 activity (percentage of control), [I]: concentration of medicinal plant extract, H: Hill coefficient.

#### 4.7.2. IC_50_ Curve-Shift for TDI Prediction, Point Screening and Relative Quantification of Phenolic Compound

The difference between the calculated IC_50_ values for pre-incubation with or without NADPH for each medicinal plant extract and diagnostic inhibitor was compared [56]. A ratio of IC_50_ value for pre-incubation without NADPH (−IC_50_) and pre-incubation with IC_50_ with NADPH (+IC_50_) was then calculated to establish the fold-shift (−IC_50_/+IC_50_). Medicinal plant extracts that showed an IC_50_ fold-shift of ≥1.5 were thus classified as TDI and those below classified as non-TDI based on the recommendation of Berry and Zhang [39]. For single point NADPH inhibition screening, a bar graph was plotted showing the percentage residual activity with the concentration of extracts with or without NADPH. The percent remaining activity was compared using the unpaired *t*-test with *p* < 0.05 considered significant. Relative quantification of metabolite profiles after UPLC-MS was performed using XCMS data analysis software.

#### 4.7.3. Prediction of *in Vivo* Herb-Drug Interaction for IC_50_

The assumptions to risk rank the HDI potential of all extracts used in this study was done according to the method of Awortwe *et al.* [57]. Percentage yield of each extract was calculated from amounts extracted from each herbal plant material. To predict the likelihood of medicinal plant extracts interacting with hepatic CYP2B6, two assumptions were made: firstly, that extracts were completely soluble in the human Gastro intestinal tract (GIT) fluid volume of 250 mL. The commonly used dose of the various extracts was estimated to be 200–400 mg. Based on the recommended dose used in humans, an estimated concentration per dose in GIT depending on the % yield was calculated; Secondly, that adequate amount of each extract enters the hepatic portal vein to interact with CYP2B6, if the IC_50_ values were lower than the estimated amount in the GIT.

## 5. Conclusions

We have shown that some of the medicinal plant extracts that have been used traditionally to cure different diseases may need to be carefully considered in the era of HAART treatment, especially when considering regimens containing EFV and NVP because of the effects of these herbal treatments on CYP2B6, an enzyme that is necessary in the deposition of many therapeutically used drugs.
